# Clinical pilot study to evaluate the neovaginal PACIENA prosthesis® for vaginoplasty without skin grafts in women with vaginal agenesis

**DOI:** 10.1186/s12905-019-0841-z

**Published:** 2019-11-26

**Authors:** Pedro Acién, Francisco J. Nohales-Alfonso, Maria-Luisa Sánchez-Ferrer, Miguel Sánchez-Lozano, Victoria Navarro-Lillo, Maribel Acién

**Affiliations:** 1 Obstetrics and Gynecologic Service, San Juan University Hospital and Department/Division of Gynecology, Miguel Hernández University, Campus of San Juan, San Juan, 03550 Alicante, Spain; 2 Obstetrics and Gynecology Service, “La Fe” University Hospital, 46009 Valencia, Spain; 3 Obstetrics and Gynecology Service, “Virgen de la Arrixaca” University Hospital and Institute for Biomedical Research of Murcia, IMIB-Arrixaca, Murcia, Spain; 4 Department of Mechanical Engineering and Energy, Miguel Hernández University, Campus of Elche, 03202 Elche, Alicante Spain; 5 Present Address: Obstetrics and Gynecologic Service, Elda General Hospital, 03600 Elda, Alicante Spain

**Keywords:** Vaginal agenesis, Vaginoplasty, Neovaginal prosthesis, Rokitansky syndrome

## Abstract

**Background:**

To evaluate the feasibility and clinical outcomes of vaginoplasties using a neovaginal polylactic acid prosthesis made with 3-dimensional (3D) printing technology as an intraneovaginal mould.

**Methods:**

This was an interventionist, prospective, and multicentre clinical pilot investigation of a sanitary product (PACIENA prosthesis®) aiming to recruit and operate on 8 patients over 6 months with a follow-up period of 6 months. Only six patients with Rokitansky syndrome and one patient with Morris syndrome (7 patients in total) were operated on in two university hospitals: “La Fe”, Valencia (H1) and “Arrixaca”, Murcia (H2). *Interventions*: Extensive surgical dissection of a defined space between the urethra and bladder in the front and of the rectum in the back as well as insertion of the PACIENA prosthesis® covered with Interceed® were performed. After 12 days, the prosthesis was changed to the silicone-covered version for daily application.

**Results:**

In the 6 patients with Rokitansky syndrome (86%), the primary endpoint (satisfactory vaginal outcome in terms of appearance, function, and sensation without relevant additional morbidity) was achieved, although only 2 patients (28%) were sexually active at the end of 6 months of follow-up. The patient with Morris syndrome withdrew from the study after 1 month. Patients without bacterial colonization showed positive Schiller tests at 1 month, and subsequent biopsies showed adequate keratinization and epidermization. Epithelization and iodopositivity were delayed in the patients who developed inflammatory granulomas.

**Conclusions:**

Good anatomical and functional results can be achieved with the PACIENA prosthesis® for vaginoplasties without skin grafts. However, adequate patient selection and education, good surgical techniques and haemostasis, postoperative support, and prevention of bacterial colonization are important.

**Trial registration:**

This clinical study was approved by the Ethical Clinical Investigation Committee of San Juan University Hospital on September 27, 2016, to be conducted in the participating centres; it was authorized by the Spanish Agency of Medicines and Health Products (AEMPS) on April 24, 2017 (exp. no. 585/16/EC), to be carried out in that hospitals.

## Background

Historically, the creation of a neovagina using a split-thickness skin graft and an inert prosthesis or synthetic mould to support the newly formed cavity (McIndoe’s technique) has been the most commonly performed vaginoplasty technique [1–4]. However, many other surgical procedures with different access pathways have also been performed (i.e., laparoscopic, laparotomic, vaginal, or combined procedures [5, 6]). On the other hand, multiple types of tissue have been applied to cover the neovaginal cavity [7]. Good results have been achieved by simply covering the prosthesis with Interceed® [8], and other authors [9, 10] have also obtained good results without using skin grafts, with evidence of squamous epithelization of the neovaginal vault. Acién et al. [11–13] suggested that by using a prosthesis made of poly-lactic acid (PLA, a biodegradable polymer derived from lactic acid often used as a scaffold in tissue engineering and regenerative medicine [14, 15] and whose contribution to epithelization has been studied by different authors [16, 17]) and designed and manufactured with 3-dimensional (3D) printing technology as a vaginal mould, skin grafting could be avoided, and vaginoplasty would be simpler, with good anatomical and functional results. We do not know of any other neovaginal prostheses for humans with the design, printing and characteristics of our PLA prosthesis.

### Study objective

This study aimed to evaluate the feasibility and clinical outcomes of vaginoplasty using a PLA prosthesis (PACIENA prosthesis®) as an intraneovaginal mould in patients with vaginal agenesis undergoing surgery following McIndoe’s technique without skin grafts.

## Methods

This study was an interventionist, prospective, and multicentre clinical investigation of a sanitary product (PACIENA prosthesis®) that aimed at recruiting and operating on 8 patients over 6 months with a follow-up period of an additional 6 months (Canadian Task Force Classification II-2). This pilot study was approved by the Ethical Clinical Investigation Committee of San Juan University Hospital on September 27, 2016, and was authorized by the Spanish Agency for Medicines and Health Products on April 24, 2017 (exp. no. 585/16/EC), for 9 participating hospitals (8 in the Community of Valencia and 1 in Murcia).

**Fig. 1:**
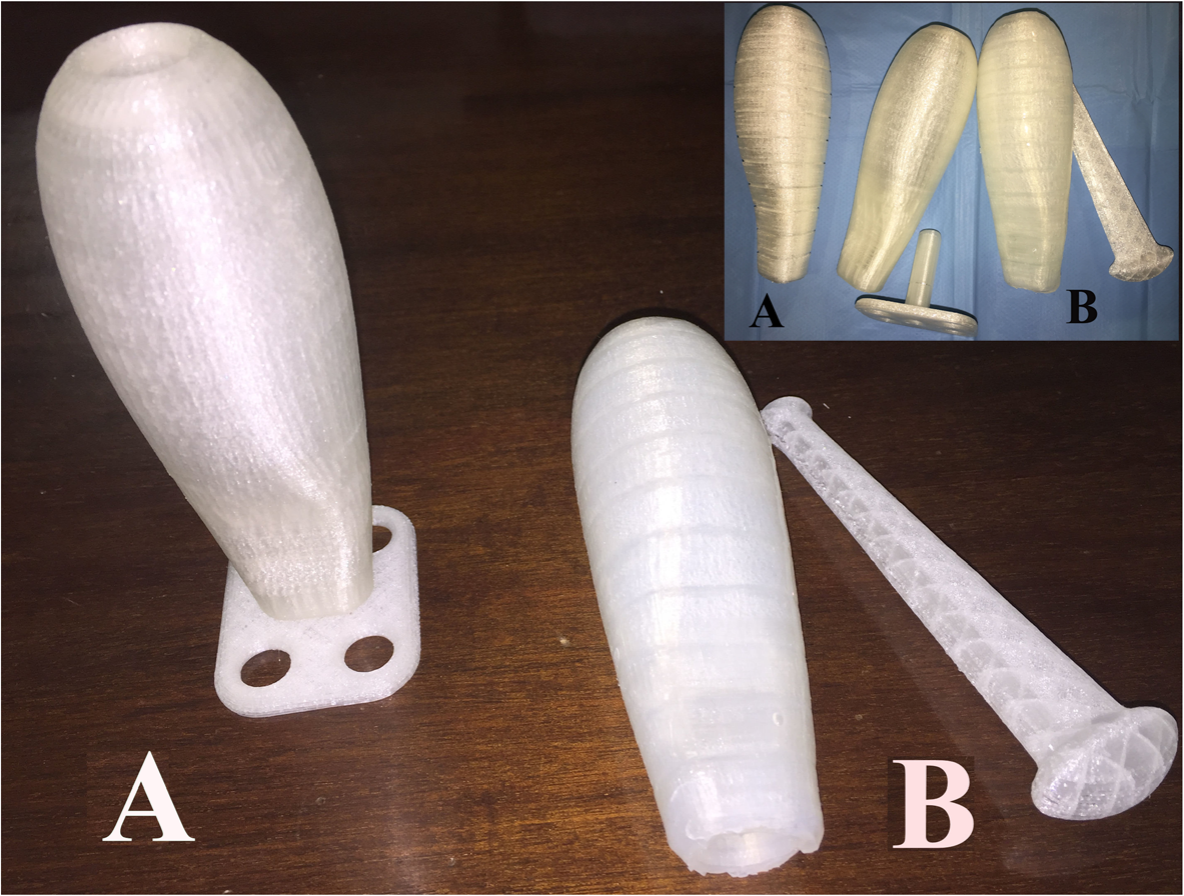
PACIENA prosthesis®. **a** Surgical prosthesis. **b** Coated silicone version of the prosthesis (the marks on the prosthesis correspond to 1 cm in length)

*The PACIENA prosthesis*® has been introduced as a new prototype of neovaginal prosthesis adapted to the vagina of normal women and has been patented (utility model U201630650, international pub. WO/2017/203076) [11–13]. The basic and novel features of this new prototype of prosthesis for neovaginas were described in [12]. This prosthesis is 130 mm long, and the diameter shows a progressive decrease from a maximum value of 38 mm up to 20 mm at the lower end to be located in the introitus; there is a recess in the lower front part, which is provided for urethral protection. The prosthesis is hollow to minimize weight and to ensure drainage through holes at both ends. A removable plate is also provided to be attached at the lower end, with holes to allow for the fixing of fastening tapes or belts. The prosthesis is made of PLA, a biocompatible material often used as a scaffold in tissue engineering and regenerative medicine applications, whose properties related to epithelial regeneration in different applications have been reported in the literature [14–17]; the use of PLA also allows customized design and manufacturing with 3-dimensional (3D) printing technology. A prosthesis that is the same shape but is covered with silicone with a smoother surface was prepared as a maintenance device (see Fig. 1).

### Inclusion criteria

1) vaginal agenesis due to MRKH (or Rokitansky) syndrome or CAIS (Morris syndrome) in women who were willing to undergo surgery for neovagina creation (McIndoe’s technique without skin grafts) after rejection or failure of previous self-dilation attempts, 2) adult age or parental authorization for younger patients, and 3) provision of written informed consent.

### Exclusion criteria

1) presence of medical illness (metabolopathy, cardiovascular, coagulation, breathing difficulties, intestinal disease) that prohibits surgery or compromises the surgical results, 2) lack of parental authorization in younger patients, and 3) a previous neovaginal surgery attempt.

### Research plan and procedures

The patients were evaluated before surgery: karyotype analysis, hormonal profile test, abdominal or transrectal ultrasound and eventually computed tomography or magnetic resonance imaging, complete physical examination, and gynaecological and mammary assessments. The diagnosis of vaginal agenesis due to Rokitansky syndrome or Morris syndrome was confirmed before surgery. The procedure consisted of the use of a prosthesis (stent or mould) that was temporarily introduced into the defined space between the bladder and the rectum of women with vaginal agenesis through the surgical intervention known as McIndoe’s technique in order to create an artificial vaginal cavity or neovagina with a suitable luminal diameter. Antibiotic cream (betametasone and gentamicin) was applied on the introduced prosthesis, and the prosthesis was covered with Interceed® mesh (an oxidized regenerated cellulose-absorbable adhesion barrier). After placing the prosthesis in the appropriate position (recess in front, suburethral), urinary flow (Foley® urethral catheter), vital signs, and blood loss were assessed, and finally, the prosthesis was fixed in place using fastening tapes or a belt. The bladder catheter was removed after 2 days, spontaneous micturition was assessed, and the patient was eventually discharged from the hospital. Then, 10–12 days later, the prosthesis was removed and replaced with the silicone-covered version. The second device was self-placed and worn daily during 6 months of follow-up and until repeated sexual intercourse occurred for a decreasing number of hours (from 20 h/day at the beginning to 6–8 h/night after 4 months).

### Outcomes

A) Primary endpoint: to achieve the formation of a satisfactory vagina in terms of appearance, function, and sensation without relevant additional morbidity. B) Secondary endpoints: 1) surgical duration, bleeding, and complications; 2) hospitalization period; 3) neovaginal length, culture, and epithelization (Schiller test and biopsy); and 4) time lapse of and satisfaction with sexual intercourse (Rosen’s Female Sexual Function Index [FSFI] [18]).

### Safety and adverse effects

Adverse effects were defined as the presence of A) stenosis due to neovaginal wall retraction, B) neovaginal fundus granulomas, C) recto-vaginal or bladder-urethra-vaginal fistulae, and D) necrosis due to compression of the urethral wall. In this study, patients with a positive vaginal culture for *Pseudomonas aeruginosa* were also included and analysed, although there were no signs of clinical infection, and additional cultures were performed after the clinical study on used and unused prostheses.

### Statistical analysis

Sample size: Due to the low incidence of this pathology, we intended to conduct a pilot study recruiting 8 patients, with an inclusion period from May to November 2017 and a follow-up period of an additional 6 months; however, only 7 patients were recruited within the authorized period. Statistical study: All data were introduced and analysed using SPSS version 25.0 (IBM, Madrid, Spain). As the number of cases was low, the main qualitative and quantitative variables were directly included in the tables, although several data are also expressed as percentages, means ± standard deviations (SDs), and 95% confidence intervals (CIs), if applicable.

## Results

Seven patients with vaginal agenesis were ultimately included during the recruitment period between May and November 2017. Of these patients, 6 had been diagnosed with MRKH (or Rokitansky) syndrome, and 1 patient was confirmed to have Morris syndrome. The hormonal profile of the 6 MRKH cases was as follows: FSH, 5.6 ± 2.8 mUI/mL; LH, 11.6 ± 14.4 mUI/mL; oestradiol, 73.4 ± 33.5 pg/mL; TSH, 2.3 ± 1.2 μUI/mL; PRL, 25 ± 13.3 ng/mL; and testosterone, 0.4 ± 0.23 ng/mL.

The age, diagnostic profile, and clinical characteristics of the patients are shown in Table 1. Patients c3 and c4 were operated on at the University Hospital “Virgen de la Arrixaca” (H2) in Murcia, whereas the other 5 patients were operated on at the University and Polyclinic Hospital “La Fe” (H1) in Valencia. All patients were examined, operated on, and evaluated following the protocol (with some variations as decided by the researcher in the intervention hospital); however, patient c6 with Morris syndrome and previous psychological instability refused the use of the silicone-covered maintenance device and refused to participate in the study in the second month. In the 6 patients with Rokitansky syndrome (86%), the primary endpoint was achieved, although only 2 of them (28%) had repeated and satisfactory sexual relations during the period of study. The other 4 did not have a sexual partner at that time. The mean age was 21 ± 3.3 (95% CI 17.9–24.1) years but varied between 19.2 ± 1.4 years (H1) and 25.5 ± 0.7 years (H2).

**Table 1:** Patient profiles

Case	Hospital	Age (range)	Diagnosis	Other anomalies	Karyotype	Urological anomalies	Uterus/ovaries	Weight (kg)	Height (cm)	BMI (kg/m^2^)
C1	H1	< 18	MRKH syndrome	Umbilical hernia	–	No	Absent/both normal	50	157	20
C2	H1	(18–21)	MRKH syndrome	–	46XX	No	Absent/both normal	51	165	18.2
C3	H2	(22–25)	MRKH syndrome	Scoliosis (op), hypoplasia MR	46XX	No	Rudimentary/both normal	51	160	19.9
C4	H2	(> 25)	MRKH syndrome	Congenital anomalies, “cat eye syndrome”	47XX + mar [18], partial trisomy 22q11.2 in mosaic	Reflux VU (op), normal kidneys	Absent/both normal	60.1	161	23.2
C5	H1	(18–21)	MRKH syndrome	Renal hypoplasia	46XX	Right renal hypoplasia, previous left nephrectomy and kidney transplant	Absent/both normal	63.3	154	26.8
C6	H1	(18–21)	Morris syndrome	Gonadectomy in childhood, Tietze syndrome	46XY	No	Absent/both absent	74	171	25
C7	H1	(18–21)	MRKH syndrome	Scoliosis, low back pain	46XX	No	Absent/both normal	58.2	155	24.3

*H1* University and Polyclinic Hospital “La Fe” in Valencia, *H2* University Hospital “Virgen de la Arrixaca” in Murcia; *MRKH* Mayer-Rokitansky-Kuster-Hauser; *BMI* body mass index, *Op* operation, *MR* magnetic resonance, *VU* vesico-ureteral

**Fig. 2:**
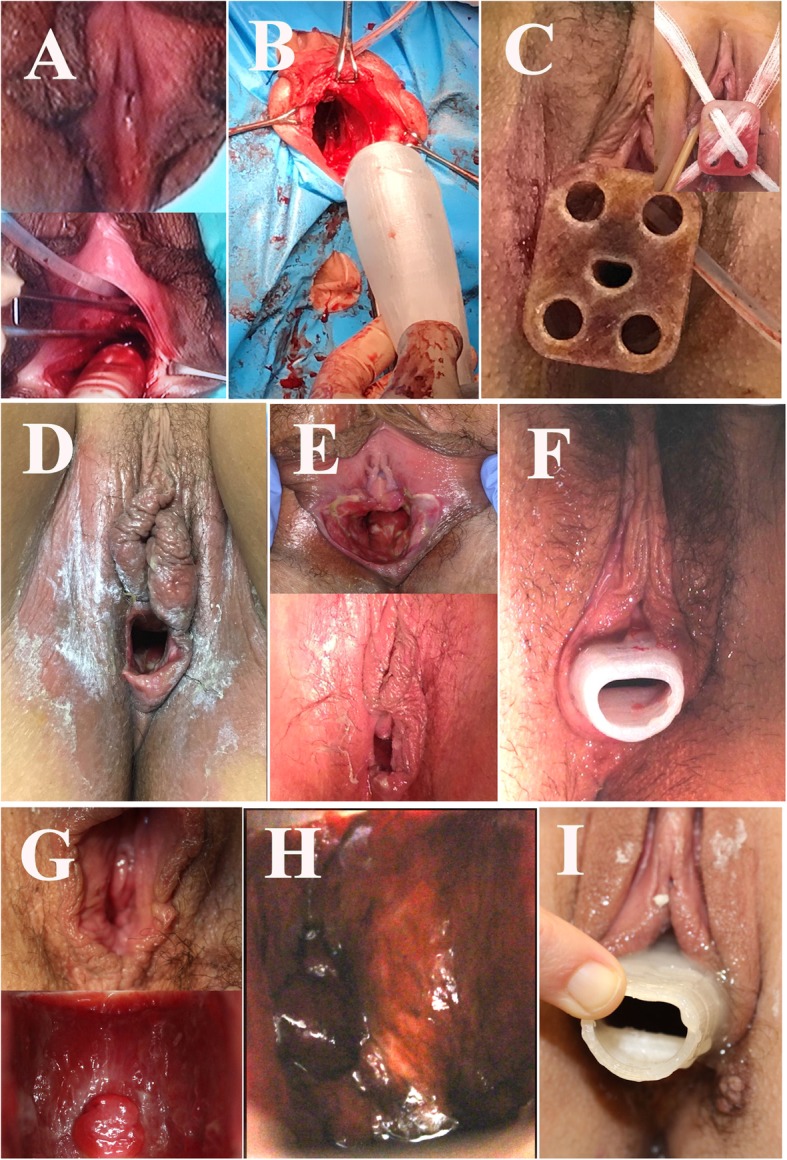
Images of the sequence of observations in the studied cases. **a** Before and at the start of the operation in c5. **b** Introduction of the Interceed®-covered prosthesis in c2. **c** Prosthesis introduced in c3 with the adapted fixation plate and cross bandages for prosthesis support. **d**. After extracting the surgical prosthesis at 12 days in c7. **e** State and extraction of the surgical prosthesis at 12 days in c5. **f** With the silicone prosthesis after the change at 12 days in c1. **g** State and Schiller’s test at 3.5 months in c3. **h** Schiller’s test at 3.5 months in c4. **i** With the silicone prosthesis at 3.5 months in c3

The surgery duration, complications, number of hospitalization days, Schiller test results, neovaginal length, culture results, and eventual sexual intercourse results are presented in Table 2. The duration of the intervention in the 7 patients was 49.3 ± 6.1 (95% CI 43.7–54.9) min (51 ± 5 min in H1 and 45 ± 7 min in H2). The length of hospital admission (7.4 ± 6.6 days, 95% CI 1.3–13.5 days) varied depending on the hospital (9.6 ± 6.7 days in H1 versus only 2 ± 0 days in H2). The mean neovaginal length at 1 and 4 months was 9 ± 1.4 (95% CI 7.6–10.2) cm and 8.7 ± 1.6 (95% CI 7–10.4) cm, respectively, remaining similar at the 6-month follow-up (8.5 ± 0.8 CI 7.6–9.4). Figure 2 shows some images of the sequence of observations in the studied cases. However, it is remarkable that the evolution and results differed between the 2 hospitals where the patients attended, as follows:

**Table 2:** Post-surgical follow-up

Case	Hospital	Date of surgery	Duration (min)	Complications	Length of admission (days)	Change of prosthesis and culture	Evaluationat 1 month			Evaluation at 3–4 months				Evaluation at 6 months			
							Sc T	Vl (cm)	Cu	Sc T	Vl (cm)	Cu	Ic	Sc T	Vl (cm)	Cu	Ic
C1	H1	June- 2017	50	Postoperative discomfort, epidural analgesia	5	D12, smaller prosthesis, PA	–	9	PA	At 3 months: areas (+), granulomas	9	At 3 months: PA	No	(+)	8	–	Yes, FSFI = 31,8
C2	H1	June-2017	50	Bleeding, pelvic haematoma	14	D12, haematoma in resolution, smaller prosthesis	–	8	PA	At 3 months: weak iodine areas, granulomas	11	PA, granulomas	No	(+/−), Granulomas	8	PA	No
C3	H2	October-2017	40	No	2	D12	(+)	10	(−)	(+)	8	(−)	No	(+)	8	–	No
C4	H2	October-2017	50	No	2	D12	(+)	11	(−)	(+)	10	(−)	Yes, repeated and satisfactory	(+)	10	–	Yes, FSFI = 32,1
C5	H1	Nov-2017	60	Moderate blood loss	3	D12, smaller prosthesis (25 mm)	–	9	PA	(+) Iodine areas, polyp	8.5	PA, biopsy: inflammatory granulation tissue	No	(+)	8	–	No
C6	H1	Nov-2017	45	Postoperative denial	19	D12, smaller prosthesis 25 mm	Left the study, no prosthesis	6,5	*Escherichia coli*	–	–	–	–	–	–	–	–
C7	H1	Nov-2017	50	No	7	D12, smaller prosthesis (25 mm)	–	9	–	(+) iodine areas, exeresis of granulomas	9	PA	No	(+), Granulomas	9	–	No

*Sc T* Schiller test, *Vl* neovaginal length, *Cu* vaginal cultures, *Ic* intercourse, *D* day; *PA Pseudomonas aeruginosa*, *FSFI* Female Sexual Function Index

**Fig. 3:**
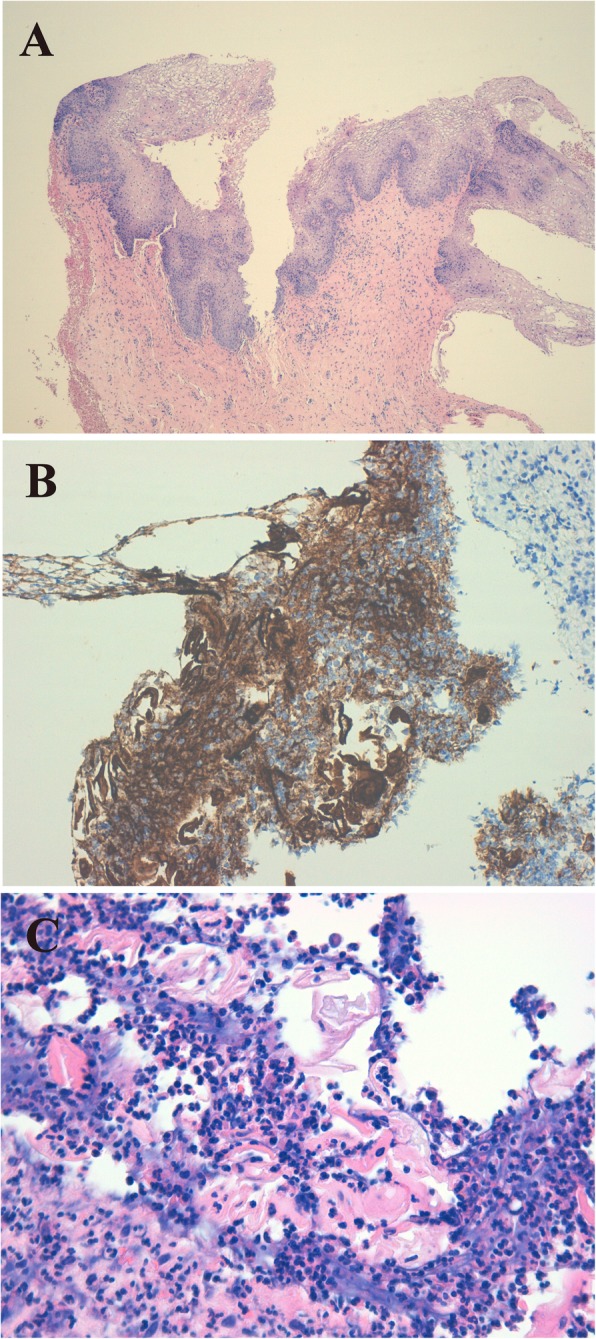
Vaginal biopsy at 6 months. **a** Well-structured, mature squamous epithelium (haematoxylin-eosin [H-E], × 40) in c4. **b** Intense staining for cytokeratin AE1-AE3 (CKAE1-AE3) on the surface of the fragment, which helps in recognizing the epithelium at that level and with greater magnification to see the epithelial positivity to cytokeratins (CKAE1-AE3, × 200). **c** Among abundant inflammatory polymorphic cells, the presence of keratin sheets (dyed more homogeneous pink) can be estimated. With greater magnification, the keratin sheets are more evident (H-E, × 400)

- Patients c1 and c2 were operated on in H1. In patient c1, due to postoperative discomfort, epidural anaesthesia was maintained, and the patient remained hospitalized until the 5th day. In patient c2, slight bleeding occurred during surgery, and postoperatively, the patient developed a pelvic haematoma and remained hospitalized for 14 days. Both patients experienced difficulties with the introduction of the silicone-covered maintenance prosthesis upon the first attempt; thus, they were given a thinner model that was later changed to the standard-sized version. In both patients, the cultures were repeatedly positive for *P. aeruginosa*. At 2 and 3 months, patched areas were observed in the Schiller test, and fundal granulomas were detected. The requirement of daily prosthesis replacement was not completely fulfilled, but at 6 months, the Schiller test was positive in both patients, and patient c1 maintained repeated and satisfactory sexual intercourse, with FSFI = 31.8 (max 36, normal mean values 27.5 ± 5.6 in [18]).

- Patients c3 and c4 attended and were operated on in H2, and they were discharged from the hospital on the 2nd day. One month after surgery, both neovaginas had a good appearance, the cultures were negative, and the Schiller test was completely positive. Patient c4 began regular sexual intercourse during the 4th month with mild dyspareunia but with a good FSFI at 6 months (32.1/36). Vaginal biopsies showed adequate keratinization and epidermization in both patients (see Fig. 3).

- Patients c5, c6, and c7 were operated on in H1. Patient c5 had moderate blood loss during the intervention but was discharged after 3 days, whereas patient c6 adopted a negative attitude postoperatively and remained hospitalized for 19 days. All patients had difficulties with the introduction of the silicone-covered maintenance prosthesis upon the first attempt; thus, they were given a thinner model that was later changed to the standard-sized version. However, the requirement of daily prosthesis replacement was not completely fulfilled. In patients c5 and c7, the cultures were positive for *P. aeruginosa*, the Schiller test showed patched areas, and fundal granulomas were present requiring subsequent exeresis. Patient c6 dropped out of the study. None of these patients had sexual intercourse during the 6-month follow-up period.

The results of the aerobic cultures taken from the prostheses, as an additional post-clinical study evaluation, are presented in Table 3. Cultures of both unused PACIENA prosthesis® versions showed growth of standard skin flora bacteria (coagulase-negative Staphylococcus). In the prosthesis culture of patient c4, after use, enteral flora were detected. However, the cultures of both used prostheses from patients c2 and c7 presented abundant *P. aeruginosa* that continued to be detected after liquid sterilization methods.

**Table 3:** Additional post-clinical study microbiological studies

Studied prosthesis	Results of aerobic culture	Repeated cultivation after Instrunet© 30 min
Unused surgical prosthesis	Habitual flora of the skin (St.c.n)	–
Unused silicone prosthesis	Habitual flora of the skin (St.c.n)	–
Silicone prosthesis used in c4 (H2)	Enteric flora, no *P. aeruginosa*	–
Silicone prosthesis used in c2 (H1)	*P. aeruginosa*	*P. aeruginosa*
Silicone prosthesis used in c7 (H1)	*P. aeruginosa*	*P. aeruginosa*

*P. Pseudomonas*, *St.c.n* coagulase-negative *Staphylococcus*

## Discussion

Our study shows that neovaginal formation and epidermization with the performance of vaginoplasty following McIndoe’s technique without the use of skin grafts and with the use of a PLA prosthesis, covered with Interceed®, as an intraneovaginal mould can be achieved with good anatomic and functional results, especially if other adverse effects (such as hospital bacterial colonization) can be alleviated. In this sense, the use of the PACIENA prosthesis® has not been associated with any complications.

The great simplicity of the operation owing to the avoidance of skin grafting and related surgery, the shortened hospital stay and the good anatomical and functional results in patients who adequately maintain the use of the silicone-covered prosthesis suggest that our vaginoplasty method using the PACIENA prosthesis may be recommended for neovaginal creation in patients with vaginal agenesis.

The well-established techniques used for vaginoplasty include grafting with split-thickness skin [1–4], peritoneum [19], bowel, free jejunal autograft, ileum [20], or sigmoid colon [21]. The Vecchietti technique has been used as a less-invasive approach to surgical vaginoplasty or as modified technique of Brucker, Rall, and Wallwiener [5, 6] using vagino-abdominal blunt perforation without vesicorectal tunnelling. Other less-invasive methods have been proposed, including Creatsas vaginoplasty (modified Williams method) [22], the labial and vestibular flap method [23], and the use of a small graft of perineal skin at the introitus to line the distal posterior aspect of the newly created vagina [24, 25]. In gender reassignment surgery, the most commonly used method is penile inversion vaginoplasty with scrotal flaps [26], sometimes combined with autologous buccal micro-mucosa free grafts [27]. However, several techniques have been attempted to cover (and epithelize) the newly formed vagina. Dhall [28] used a human amnion graft, whereas Zhang et al. [7] and Zhu et al. [29] used a biological mesh (tissue-engineered biomaterial graft). Other authors have used Interceed® [8], or the placement of this mesh between the stent and a skin graft (meshed) and thus reducing the size of the graft, while maintaining good results related to neovaginal epithelization [30].

Nevertheless, all these techniques involve dissecting the vesicorectal space and inserting a stent into the neovagina to help the adherence of the graft or to maintain patency. For such purposes, some authors have proposed the use of a silicone mould of different dimensions or even a customized mould [31, 32]. Other authors used stents of different materials (foam rubber, wood, plastic, glass, Teflon, Dexon, vacuum expandable condom, a simple syringe or a polyethylene bag [7, 30, 33–36]), many of which are not anatomically designed. These stents are usually hand-made, using weighty and strong materials, and can produce bedsores on the recto-vaginal septum or necrosis on the urethra and hypospadias. Therefore, the stent or neovaginal mould might be important for achieving good anatomical and functional results.

The shape and dimensions of the PACIENA prosthesis® are adapted to the normal vagina, and it is made of PLA, which is a biocompatible material used in several biomedical applications and whose effect on tissue growth has been reported in different scenarios [14–17, 37–39]. For example, Sharma et al. [17] developed a skin substrate made of PLA scaffold with minced skin grafts; skin cells were shown to migrate along the fibres of the scaffold, new collagen was formed, and epithelial and stromal cells were confirmed by immunohistochemistry and scanning electron microscopy [17].

In our pilot study, we used an Interceed® mesh to cover the PLA prosthesis as a barrier to prevent the adhesion of the newly created neovaginal tissue to the prosthesis and that has to be removed after several days. As a result, it might have been the combination of the geometry of the prosthesis, the anatomical design, the manufacturing of the PLA, and the Interceed® covering that has led to satisfactory results avoiding the need for skin grafts. In addition, the design and shape of the prosthesis (wider in the fundus with a decreasing diameter at the bottom end) allow self-support by the perineal muscles without needing to use other fastening aids or suturing of the labia.

Therefore, if a prosthesis has the adequate dimensions and the basic and novel features of the PACIENA prosthesis®, then good anatomical and functional results and adequate epidermization can be expected, similar to what occurs in normal vaginal embryology from the urogenital sinus [40], which in an adult woman is the vaginal introitus.

### Clinical relevance

The following aspects suggest that this technique could be the most suitable method for vaginoplasty in the congenital absence of a vagina and in other situations, including gender reassignment surgery, ablation, or surgery for an acquired pathology (synechiae): a simple technique requiring only the dissection and opening of the vesicorectal space, making the procedure minimally invasive; the absence of complications related to skin grafting or to the use of a hard and heavy prosthesis; and the anatomical design of the PLA prosthesis using 3D technology, with a material composition that favours tissue growth.

### Study limitations and unexpected findings

This pilot study was designed and authorized to recruit 8 patients for evaluation over 6 months (with an additional 6 months of follow-up); however, only 7 patients could be included. Only 6 patients (all with Rokitansky syndrome) completed the 6-month use of the prosthesis and the follow-up. All patients treated in H1 were positive for *P. aeruginosa*, which could have induced inflammation and granulomas as well as worsened the results. Some influence of age, psychological preparation before surgery, or surgical technique might also be present.

## Conclusions

Good anatomical and likely good functional results can be achieved with the PACIENA prosthesis® for neovaginal creation (vaginoplasty) following a modification of McIndoe’s technique to avoid skin grafting. However, a good surgical technique and haemostasis, postoperative support, and the prevention of bacterial colonization are also important. Moreover, adequate patient selection with appropriate education, prosthesis self-replacement recommendations, and psychological support before and after surgery also seem fundamental.

## Data Availability

PA had full access to all study data and takes responsibility for the integrity of the data and the accuracy of the data analysis. Statement of prior presentation or publications and/or abstract/poster presentation: No prior presentation, including the cases reported. Yes on prosthesis, References 11–13.

## Abbreviations

3D: 3-dimensional printing technology
CAIS: Complete androgenic insensibility syndrome (or Morris syndrome)
FSFI: Female Sexual Function Index
H1: University and Polyclinic Hospital “La Fe” in Valencia
H2: University Hospital “Virgen de la Arrixaca” in Murcia
MRKH: Mayer-Rokitansky-Kuster-Hauser (or Rokitansky) syndrome
PLA: Poly-Lactic Acid
